# *Caenorhabditis elegans* as Model System in Pharmacology and Toxicology: Effects of Flavonoids on Redox-Sensitive Signalling Pathways and Ageing

**DOI:** 10.1155/2014/920398

**Published:** 2014-04-30

**Authors:** Karoline Koch, Susannah Havermann, Christian Büchter, Wim Wätjen

**Affiliations:** Institute of Agricultural and Nutritional Sciences, Martin-Luther-Universität Halle-Wittenberg, Weinbergweg 22 (Biozentrum), 06120 Halle, Germany

## Abstract

Flavonoids are secondary plant compounds that mediate diverse biological activities, for example, by scavenging free radicals and modulating intracellular signalling pathways. It has been shown in various studies that distinct flavonoid compounds enhance stress resistance and even prolong the life span of organisms. In the last years the model organism *C. elegans* has gained increasing importance in pharmacological and toxicological sciences due to the availability of various genetically modified nematode strains, the simplicity of modulating genes by RNAi, and the relatively short life span. Several studies have been performed demonstrating that secondary plant compounds influence ageing, stress resistance, and distinct signalling pathways in the nematode. Here we present an overview of the modulating effects of different flavonoids on oxidative stress, redox-sensitive signalling pathways, and life span in *C. elegans* introducing the usability of this model system for pharmacological and toxicological research.

## 1. Flavonoids

Flavonoids are polyphenolic compounds that occur ubiquitously in fruit, vegetables, grains, nuts, tea, and wine (reviewed by [1, 2]). The basic structure of flavonoids consists of an O-heterocyclic ring (B) fused to an aromatic ring (C) with a third ring system (A) attached at C2 of the heterocyclic ring (=phenylbenzopyrones). Up to now, over 6000 flavonoids have been identified.

A high number of flavonoids have been shown to be potent antioxidants due to their ability to donate electrons and the stabilisation of oxidized flavonoid species (semiquinone radical). Moreover, flavonoids form complexes with redox-active heavy metal ions, for example, Fe^2+^ and Cu^2+^ that are involved in Fenton-like reactions [3]. The antioxidant potential of flavonoids has been believed to be mediated by their biological actions for a long time, but from today's point of view it is by no means clear that other mechanisms of action contribute to their overall effect and are even more important than the radical scavenging properties. Flavonoids possess a remarkable spectrum of biochemical and pharmacological activities affecting basic cell functions (reviewed by [4]).

For example, mitogen-activated protein kinases (MAPK), major regulators of cell growth, proliferation, differentiation, and death, were shown to be modulated by these plant compounds. Specific flavonoids were shown to inhibit extracellular signal-related kinase (ERK-1/2), c-Jun amino-terminal kinase (JNK1/2), and p38-MAP kinase in cancer cell lines and stimulated immune cells [5, 6]. Several flavonoids were also found to inhibit the nuclear factor-*κ*B (NF-*κ*B) signalling pathway which is involved in inflammatory, immune, and stress response as well as in apoptosis and proliferation by activation of I*κ*B-kinase or blocking NF-*κ*B-DNA association [7, 8]. Consequently, suppression of NF-*κ*B signalling by flavonoids resulted in the inhibition of proinflammatory enzymes like phospholipase A_2_ (PLA_2_), lipoxygenase (LOX), and cyclooxygenase (COX) which are important targets in pharmacological sciences [9]. Flavonoids and isoflavonoids were found to influence the NO production by modulating the gene and protein expression of nitric oxide synthase (NOS) isoforms. Hämäläinen et al. [10] showed that flavone, daidzein, genistein, isorhamnetin, kaempferol, and quercetin inhibited the mRNA and protein expression of the inducible NOS in lipopolysaccharide stimulated murine J774 macrophages which was associated with a decreased activation of NF-*κ*B. Some of these compounds were also found to inhibit the activation of the signal transducer and activator of transcription 1 (STAT-1). On the other hand the green tea flavonoid epigallocatechin-3-gallate (EGCG) was shown to increase endothelial nitric oxide synthase (eNOS) activity in a PI3K/PKB-dependent manner leading to vasodilation of rat aortic rings with the potential to protect against cardiovascular diseases [11].

Several molecular targets are discussed to explain the cytotoxicity of some flavonoids, for example, inhibition of protein kinase C [12], phosphorylase kinase [13], AMP phosphodiesterase [14], DNA topoisomerases [15, 16], glutathione S-transferases [17] leading to, for example, disruptions in cell cycle followed by apoptotic or necrotic events. An induction of apoptosis by flavonoids was reported for example, by Wei et al. [18] and Richter et al. [19].

Further on, diverse mechanistic studies have reported that flavonoids directly interfere with cell cycle regulation. For example, gallated catechins were reported to induce cell cycle arrest due to the suppression of cyclin D, cyclin E, and cyclin-dependent kinases (CDK) 1 and 4 protein expression [20, 21].

Besides the influence on basic cell functions, flavonoids are considered to exert chemoprotective effects by a modulation of the arylhydrocarbon-receptor (antagonists or weak agonists) which mediates the toxicity of halogenated aromatic hydrocarbons and polycyclic aromatic hydrocarbons [22, 23].

Because of the broad activity spectrum of flavonoids in diverse cell signalling pathways these phytochemicals are discussed to be protective against several diseases like cancer, obesity and diabetes [24], rheumatoid arthritis [25], and inflammatory diseases [26]. In fact, consumption of a diet rich in flavonoids is associated with beneficial effects to human health. In a prospective cohort study it has been shown that flavonoid intake is negatively associated with the risk of mortality from coronary heart disease (reviewed by [27]). Associations between the dietary intake of flavonoids and the incidences of a variety of cancers have also been studied in numerous prospective and case-control studies. Significant associations were observed for the incidence of, for example, oral, gastrointestinal, colorectal, liver, ovarian, endometrial, prostate, and breast cancer (reviewed by [28]). Consumption of flavonoid rich food and beverages like wine, chocolate, and tea was associated with an improved cognitive performance in the elderly, too [29]. Noticeable, these phytochemicals are claimed to protect mainly against diseases that occur in an age-dependent manner and proceed chronically. In societies with an increasing proportion of elderly and consequently rising incidences of age-related diseases it is important to seek for substances that may be utilized for pharmaceutical purposes in terms of prevention. Moreover it is important to understand the molecular mechanisms of ageing in order to develop strategies for modulating ageing-associated physiological changes and degenerations leading to chronic diseases. Due to the possibility to manipulate cell signalling pathways by simple biotechnological methods, the model organism* C. elegans* offers promising possibilities for studying the influence of secondary plant compounds like flavonoids on the process of ageing.

## 2. Modulation of Life Span of* C. elegans* by Flavonoids

**Figure 1:**
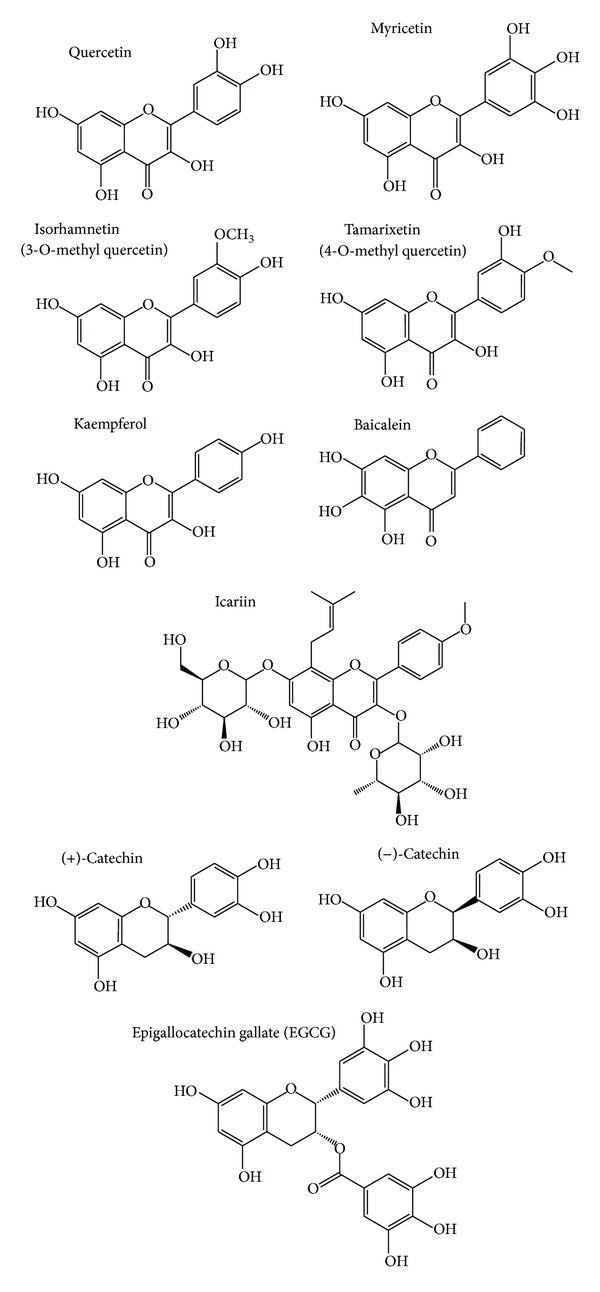
Major flavonoids studied in* C. elegans*.

Distinct flavonoids (Table 1) and flavonoid-rich plant extracts (Table 2) have been shown to modulate life span of* C. elegans*. The chemical structure of the compounds are shown in Figure 1. The prominent flavonoid quercetin (100 to 200 *μ*M) increases mean and median life span by about 6–18% and 19–21%, respectively [30–36]. Additionally, nematodes treated with quercetin showed a higher (5 to 18%) maximum life span than controls [30, 36]. Pietsch et al. [35] revealed that quercetin mediated life span extension in* C. elegans* was characterised by an inverted U-shaped dose-response curve.

Increasing hydroxylation on rings A and B of the basic flavonoid structure seems to be crucial for the longevity effect in the nematode. It has been shown that flavonoids from the flavonol type prolonged the mean life span of* C. elegans* according to their number of OH groups in the B ring in the following order: kaempferol < quercetin < myricetin [31–38]. Baicalein, containing an additional OH group in the A ring, showed the most powerful effects. This flavonoid (100 *μ*M) increased mean, median, and maximum life span by 45, 57, and 24% [39].

Methylated derivatives of quercetin were shown to increase the mean life span of worms: 4-O-methylated quercetin (tamarixetin) was more efficient in life span prolongation than a methylation at position 3 (isorhamnetin) [34]. Glucopyranosides of the flavonoid quercetin 3′-O-*β*-D-glucopyranoside (Q3′G) and quercetin 3-O-*β*-D-glucopyranoside-(4→1)-*β*-D-glucopyranoside (Q3M) increased the mean life span of* C. elegans* compared to the control. The effect was comparable with the life span prolongation of quercetin [33]. Icariin and icariside II, bioactive compounds of* Herba epimedii,* are structurally related to a 3′-O-methylkaempferol glycoside. These flavonoids were found to increase the mean life span of* C. elegans* to a higher extent compared to kaempferol (21% versus 6%) [36, 40].

Flavonoids with a flavan-3-ol structure also induced a prolongation of life span in the nematodes: Saul et al. [41] showed that catechin enhanced mean and median life span of* C. elegans* at concentrations from 100 to 800 *μ*M [41]. The most effective concentration (200 *μ*M) of the flavonoid increased mean and median life span by about 9 and 13%. In contrast, Surco-Laos et al. [42] did not observe an effect of catechin on longevity. However, epicatechin [43] and its 3′-O- and 4′-O-methylated derivatives could significantly enhance mean and maximum life span of the worms, once more indicating the importance of methylation. Similar to the quercetin derivatives, 4′-O-methylation induced a higher life span extension than 3′-O-methylation [42]. EGCG caused a mean life span expansion at high concentrations (100 *μ*M: 20% and 220 *μ*M: 14%) [43, 44] while low concentrations (under 25 *μ*M) of the flavonoid did not induce longevity effects in* C. elegans*.

Next to the single compound, flavonoid-rich plant extracts were studied in regard to their life span prolonging properties (Table 2). In this context, procyanidins from cocoa apple and blueberry extracts were shown to enhance the mean life span by 12 to 17% in the nematode [45–47]. The* Gingko biloba* extract EGb 761 being rich in flavan-3-ol glycosides increased mean, median, and maximum life span of the nematode by 10, 16, and 17% [48].

## 3. Influence of Flavonoids on the Oxidative Stress Level in* C. elegans*

**Figure 2:**
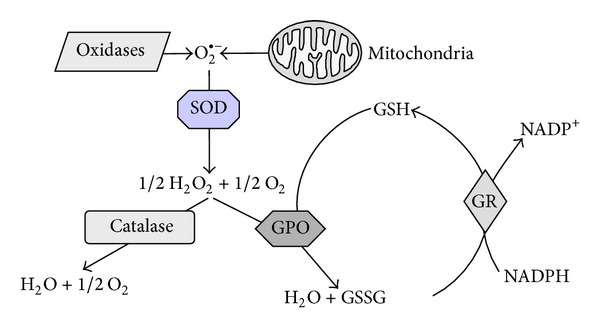
Schematic illustration of the endogenous defence system against intracellular oxidative stress. CAT: catalase, GPO: glutathione peroxidase, GR: glutathione reductase, GSH/GSSG: reduced glutathione/oxidized glutathione, SOD: superoxide dismutase.

Analysis of oxidative stress is important for pharmacological and toxicological research because reactive oxygen species (ROS) may modulate or even destroy central cellular parameters. ROS originate from exogenous sources like UV irradiation or environmental toxins, but, also from endogenous sources. The main site of intracellular ROS production is the electron transport chain in the mitochondria. Other endogenous sources of ROS are intracellular enzymatic systems like the NADPH oxidase family, lipoxygenases, cytochrome P450, and the peroxisome (reviewed by [49]). Redox-active heavy metal ions like Fe^2+^ and Cu^2+^ also lead to the generation of ROS through fenton-like reactions [50]. Since ROS generation is the physiological result of every aerobic metabolism, it seems to be necessary for the viability of the cell. Low concentrations of ROS are needed for transducing cellular signals in various processes like proliferation, apoptosis, inflammation, and immune response [51]. On the other hand, an excess of these highly reactive molecules can cause severe damage to cellular molecules resulting in lipid peroxidation, oxidation of DNA bases, as well as protein modifications leading to modified activities. Since mitochondria are especially sensitive to oxidative stress due to high concentrations of radicals like superoxide anions (O_2_^−^), an increase in ROS will disturb the mitochondrial electron transport chain resulting in an impaired energy production. Therefore ROS have to be inactivated by different endogenous antioxidant mechanisms, for example, antioxidant enzymes like superoxide dismutases (SOD), and glutathione peroxidases (GPO), and catalase (CAT), or low molecular antioxidants like glutathione (Figure 2). If the generation of ROS exceeds the antioxidant potency, accumulation of these oxidised toxic molecules in tissues leads to the phenomenon of so-called “oxidative stress” [52, 53]. On the cellular level, oxidative stress may cause the induction of apoptotic cell death or a disruption of intracellular signal transduction pathways [54].

The ageing process is associated with an increase in oxidative stress leading to impaired function and reproductivity of the cell as well as gene mutations which result in an increased likelihood for degenerative diseases and cancer [55]. In fact, oxidative stress is an important risk factor for many human diseases, for example, cardiovascular diseases [56], neurological disturbances such as morbus Alzheimer (reviewed by [57]), cancer [58], and chronic obstructive pulmonary disease [59]. Therefore substances that have the ability to reduce oxidative stress have been in the focus of pharmacological research for a long time. The use of flavonoids which were found to act as radical scavengers as well as indirect antioxidants may be a promising intervention strategy to prevent these age-associated diseases.

### 3.1. The Influence of Flavonoids on the Basal Oxidative Stress Level in* C. elegans* (Physiological Conditions)

Flavonoids have the potential to modulate intracellular oxidative stress directly by scavenging free radicals. In* C. elegans* intracellular ROS levels can be determined by the use of fluorescent probes, for example, H_2_DCF-DA, MitoTracker Red, or CM-H2XRos.

#### 3.1.1. Effects of Flavonoids on ROS Level and Oxidative Cell Damage in* C. elegans* (Direct Antioxidative Effects)

Several flavonoids were shown to reduce basal ROS levels in* C. elegans* [36, 44, 60–62].

Quercetin and the structural related flavonols myricetin, and kaempferol (each 100 *μ*M for 48 h) decreased mitochondrial ROS levels in* C. elegans* by about 70%, 60%, and 20%, whereas naringenin did not show significant effects [36]. The authors explained that the differences between the the ROS reducing capacity were due to the structural nature of the substances (Figure 1). The 3-OH group and the double-bond in the C-ring, which are missing in naringenin, seem to be necessary for the mtROS scavenging effect and the catechol function in the B ring is thought to enhance the antioxidative effect in* C. elegans*. However, the methods for detecting ROS in* C. elegans* vary: While Grünz et al. [36] analysed mitochondrial ROS accumulation (MitoTracker Red, CM-H2XRos) in the living nematode, other researchers determined the influence of flavonoids on total intracellular ROS levels (H_2_DCF-DA) by analysing sonificated nematodes. For example, Gonzáles-Manzano et al. [61] showed that 200 *μ*M of epicatechin reduced basal levels of intracellular ROS by 10% in the nematode [61]. In addition it has been observed that EGCG decreased basal H_2_O_2_ levels in* C. elegans* in a dose-dependent manner whereby the most efficient dose (220 *μ*M EGCG for 72 h) reduced H_2_O_2_ levels by about 32% [44, 60].

An increase of intracellular ROS concentrations may lead to oxidative stress associated cell damage. In order to investigate whether flavonoids are also protective against this kind of cell damage protein and lipid oxidations products were measured. To the best of our knowledge, the influence of flavonoids on DNA oxidation has not been investigated in* C. elegans *so far.

Icariside II, myricetin, quercetin and its methylated forms isorhamnetin and tamarixetin were found to protect against protein oxidation in* C. elegans* [34, 36]. Grünz et al. [36] studied the effect of different flavonoid compounds on the level of proteincarbonyls which emerge during oxidation of protein amino acid side chains or indirect modifications due to products of lipid peroxidation or glycation [63]. The author showed that 100 *μ*M of quercetin or myricetin decreased proteincarbonyl levels by 60 and 50%, respectively [36].

Cai et al. [40] investigated the influence of the bioactive flavonol-diglycoside icariside II from* Herba epimedii* on polyglutamine (polyQ) aggregation in the AM140* C. elegans* strain which expresses polyQ fused to the yellow fluorescent protein (YFP) in body wall muscle cells. Mutated proteins with an elongated GAC sequence coding for glutamine cause the neurodegenerative Huntington's disease. The aggregation of these polyQ containing proteins is induced by oxidative stress and related to the pathogenesis of the disease [64]. Therefore transgene polyQ::YFP* C. elegans* are used as a model to analyse the oxidation status of proteins [65]. 20 *μ*M of icariside II was shown to significantly decrease formation of polyQ aggregates in the worms at days 4 and 6 of adulthood compared to untreated controls [40]. The polyQ induced paralysis phenotype of the worms was also decreased by icariside II.

Moreover, Mohri-Shiomi and Garsin [65] investigated the capacity of EGCG to protect against infection mediated cell damage in* C. elegans*. 25 *μ*M of EGCG was shown to reduce (1.9-fold) polyglutamine accumulation after pathogen (*E. faecalis*, day 2) infection in AM141* C. elegans* expressing polyQ::YFP only in the intestine compared to the control. Therefore the authors suggested that EGCG protects against infection induced oxidative protein damage [65].

Another method to determine direct antioxidative effects of flavonoids in* C. elegans* is the analysis of lipid oxidation by measuring levels of lipofuscin. This autofluorescent pigment consists of oxidised cross-linked lipidperoxide products and partly oxidised proteins which cannot be degraded by the lysosome and thus accumulate within the cell in an age-dependent manner [66, 67]. In* C. elegans *lipofuscin particles can be seen throughout the gut and serve as ageing markers reflecting oxidative cell damage [68]. Flavonoids decrease the accumulation of lipofuscin pigments in* C. elegans* (Table 3) indicating that these plant compounds possess a protective effect towards age-associated oxidative cell damage [30, 35, 37, 38, 45, 69]. For example, Kampkötter et al. [30, 37] demonstrated that 100 *μ*M of quercetin (48 to 72 h) reduced lipofuscin accumulation by 40% in seven-day-old* C. elegans*. Similar results were obtained by Pietsch et al. [35] where a treatment with 200 *μ*M quercetin resulted in a 16% decrease of lipofuscin autofluorescence at day 7 of adulthood*. C. elegans* treated with kaempferol or myricetin (100 *μ*M) did also show diminished lipofuscin autofluorescence whereas rutin and fisetin did not influence lipofuscin levels in the worm [37, 38, 70]. Further on, daily administration of 220 *μ*M of EGCG for 16 days decreased lipofuscin levels by about 39% in sterile* fem-1 C. elegans* compared to the control [69]. Analogous effects were observed for blueberry polyphenols [45]. Additionally the authors measured levels of 4-Hydroxynonenal (4-HNE), a secondary oxidation product of lipids accumulating in an age-dependent manner in various species. Treatment with a flavonoid rich blueberry extract significantly reduced 4-HNE levels in the pharynx and the somatic gonads of 14-day-old* C. elegans*.

#### 3.1.2. Modulation of Antioxidant Enzymes by Flavonoids in* C. elegans* (Indirect Antioxidant Effects)

Flavonoids are able to modulate oxidative stress not just directly but also indirectly by modulating antioxidative enzymes (SOD-3, CAT, GPO, and glutathione-S-transferase (GST)). To analyse these effects, transgenic* C. elegans* were used, for example, nematodes expressing the green fluorescent protein (GFP) under the control of the promoter of these marker genes. In addition mRNA expression of antioxidative enzymes was determined.

For EGCG it has been shown that 0.1 *μ*g/mL of the flavonoid upregulates SOD-3 expression by 49% [71]. Quercetin treatment of* C. elegans* resulted in a 15% decreased expression of the antioxidant marker enzyme SOD-3 [30]. The authors proposed that the reduction of SOD-3 expression may be the result of the direct antioxidant effects of the flavonoid as it was observed that quercetin reduces ROS levels under diverse stress conditions [30, 37].

Kampkötter et al. [48] did also study the effect of the* Ginkgo biloba* extract EGb 761 on antioxidant enzyme expression levels. It was shown that this extract suppressed GST-4 protein expression by 28%. Additionally the plant extract treatment resulted in a 32% reduction of catalase transcription levels in the nematodes. These results may also reflect direct antioxidant effects of the plant extract. In conclusion, various flavonoids, depending on their structural properties, were experimentally shown to decrease oxidative stress in* C. elegans* under basal conditions.

### 3.2. Influence of Flavonoids on Oxidatively Stressed* C. elegans* (Pathophysiological Conditions)

To simulate oxidative stress the nematodes are treated with redox cyclers (e.g., juglone) or hydrogen peroxide. Another possibility is the use of mutant strains producing large amounts of ROS. For example,* mev-1(kn*)* C. elegans* are hypersensitive to oxidative stress due to a functional loss of a subunit of the enzyme succinate dehydrogenase cytochrome b (complex II in the electron transport chain) [72]. To analyse the effects of flavonoids on ROS levels, antioxidant marker enzymes and survival rates of oxidatively stressed nematodes are analysed after flavonoid or vehicle treatment.

#### 3.2.1. Effects of Flavonoids on ROS Level and Stress Resistance in Oxidatively Stressed* C. elegans* (Direct Antioxidant Effect)

EGCG [71], aspalathin [62], myricetin, quercetin, and kaempferol [36] were found to be effective in lowering ROS levels in* C. elegans*. Zhang et al. [71] demonstrated that oxidatively stressed (1 h juglone) nematodes pretreated with 0.1 *μ*g/mL EGCG (for 48 h) showed significantly lower levels of ROS than untreated controls. In addition Chen et al. [62] showed that 50 *μ*M of aspalathin decreased ROS levels by about 42% in* mev-1(kn).* Similar results were obtained for myricetin, quercetin, and kaempferol as mitochondrial ROS levels were diminished by 45, 60, and 23% in* mev-1(kn) C. elegans* [36].

Flavonoids were also reported to act as potent mediators of oxidative stress tolerance in* C. elegans*. For example, 100 *μ*M of quercetin enhanced survival times of* C. elegans* incubated with hydrogen peroxide [31] or the redox-cycler juglone [30] as chemical stressors by about 20%. In addition, Surco-Laos et al. [34] showed that 200 *μ*M of quercetin enhanced mean survival rates by 12% under juglone treatment while the methylated derivatives isorhamnetin and tamarixetin showed slightly weaker effects (9 and 10%). The authors also demonstrated that the flavonoids are more protective when oxidative stress occurs in a later stage of adulthood (6th day) than in the first day of adulthood.

Catechin, epicatechin, and its methylated derivatives increased survival rates up to about 1.4-fold [42]. In addition, EGCG enhanced survival rates of* C. elegans* about 3.9-fold [44]. Quercetin (200 *μ*M) [32] and EGCG (50 *μ*M) [44] also increased mean life span of* mev-1(kn)* mutants by 10% and 16% indicating that these flavonoids induce oxidative stress tolerance in* C. elegans* [44].

Some flavonoid rich plant extracts were shown to influence oxidative stress resistance in* C. elegans *while others did not mediate any effects. The catechol-rich Willow bark extract increased survival rates of* C. elegans* under tert-butylhydroperoxide induced oxidative stress up to 33% while the blueberry extract did not show significant effects [45]. Procyanidines from blueberris [45] or apples [47] did also not enhance mean life span of* mev-1(kn) C. elegans*. Therefore, Sunagawa et al. [47] assumed that the analysed procyanidins possess no antioxidant effect in* C. elegans *which mediates life span expansion.

#### 3.2.2. Modulation of Antioxidant Enzymes by Flavonoids in Oxidatively Stressed* C. elegans* (Indirect Antioxidant Effects)

Kampkötter et al. [37, 48, 70] studied the effects of different flavonoids on the protein expression level of the marker enzyme GST-4 under juglone-induced oxidative stress. Quercetin treatment was shown to decrease GST-4 expression by 50% in transgenic worms under oxidative stress [37]. Kaempferol, rutin, and fisetin did also mediate a 45, 15, and 10% decline of the GST-4::GFP level in* C. elegans* [37, 70]. In addition* C. elegans* showed a reduction of GST-4 expression by 35% when treated with the flavonoid-rich* Ginkgo biloba* extract EGb 761 [48].

### 3.3. Influence of Flavonoids on ROS Levels and the Stress Resistance in* C. elegans* under Thermal Stress

An increase of the temperature from 20°C (basal) to 35–37°C is lethal to* C. elegans*. This thermal stress is accompanied by an increase in ROS as well as an increase in nonfunctional proteins.

#### 3.3.1. Effects of Flavonoids on ROS Level and Stress Resistance in* C. elegans* under Thermal Stress (Direct Antioxidant Effect)

Various types of flavonoids were able to diminish oxidative stress levels induced by heat [37–39, 48, 61, 70]. For example, epicatechin treatment decreased intracellular levels of ROS by 24 to 28% [61]. In addition thermal stress induced increase of oxidative stress was reduced by 100 *μ*M of quercetin and, to a lesser extent, rutin [37]. Kaempferol, fisetin, baicalein, and myricetin did also decrease intracellular levels of ROS after 4 to 6 h of thermal stress [38, 39, 70]. Moreover 100 *μ*g/mL of the* Ginkgo biloba* extract EGb 761 decreased intracellular ROS levels in* C. elegans* by about 30% after thermal stress exposure [48].

Various types of flavonoids were also shown to enhance life span of* C. elegans* under thermal stress [31, 32, 34, 37, 41, 42, 44, 45, 48, 73]. Treatment with quercetin increased mean survival times under thermal stress by about 12% to 30% [32, 34, 37, 41]. The methylated derivatives isorharmnetin and tamarixetin showed even more powerful effects by enhancing mean survival times by 39% [34]. EGCG, catechin, epicatechin, and 3′/4′-O-methylepicatechin were shown to increase survival rates of* C. elegans* by approximately 9% to 28% [41, 42, 44]. Further on Wilson et al. [45] showed that proanthocyanidins from blueberries enhanced the survival rates 2.5-fold. The flavonoid rich* Ginkgo biloba* extract EGb 761 and taxifolin did also increase thermotolerance in* C. elegans* [48, 73].

#### 3.3.2. Modulation of Antioxidant Enzymes by Flavonoids in* C. elegans* under Thermal Stress (Indirect Antioxidant Effects)

The modulation of antioxidative enzymes during thermal stress conditions is also affected by flavonoids. Gonzáles-Manzano et al. [61] studied the influence of epicatechin on the expression levels of GPO in* C. elegans* under thermal stress. Under these conditions 200 *μ*M of epicatechin tend to diminish the expression of GPO in GPO::GFP* C. elegans* [61]. Kampkötter et al. [37, 48, 70] showed that* Ginkgo biloba* extract EGb 761 reduces GST-4 expression during thermal stress.

## 4. Modulation of Representative Redox-Sensitive Signalling Pathways

Oxidative stress results in the activation of a variety of intracellular signalling pathways in* C. elegans* resulting in an altered gene expression which determines cellular fate. Hence, several pathways induce cellular defence and adaption systems; others mediate proliferation or even apoptosis. The mitogen activated kinases ERK, JNK, and p38 as well as the Phosphatidylinositol-4,5-bisphosphate 3-kinase (PI3K)/Akt pathway belong to the major stress activated signalling pathways. While ERK and AKT exert cytoprotection, JNK and p38 are commonly associated with programmed cell death upon oxidative stress (review by [74]).

To analyse the molecular effects of flavonoids, the investigation of signalling pathways is crucial. In this review we show studies on modulation of selected cellular pathways involved in stress response and ageing by flavonoids.  Table 4 gives an overview of the main regulatory ageing-associated genes in* C. elegans* and their mammalian homologues.

### 4.1. Insulin/IGF-I Signalling Pathway

#### 4.1.1. The Insulin/Insulin Like Growth Factor (IGF)-I Signalling Pathway

**Figure 3:**
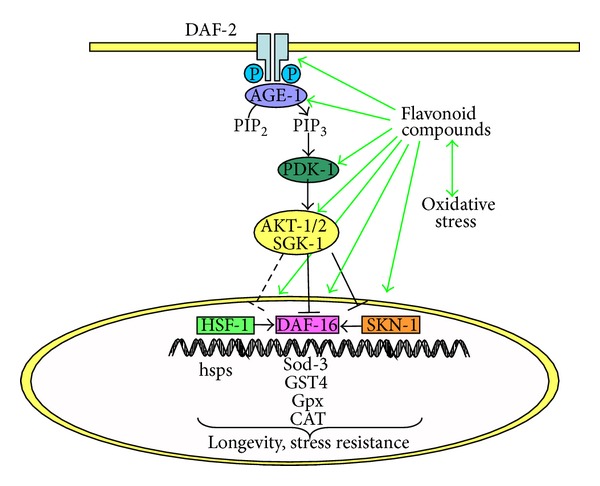
Simplified scheme of the DAF-16 signalling pathway in* C. elegans* representing molecular targets for modulation by flavonoids.

The Insulin/Insulin like growth factor (IGF-)I signalling pathway is an evolutionary conserved mechanism that influences longevity and metabolism throughout different species (reviewed in [75]). In* C. elegans* this kinase cascade is required for the formation of the dauer larval stage and ageing [76]. The initiation of the cascade depends on the phosphorylation of DAF-2, the* C. elegans* homologue for the Insulin/IGF-1 receptor, which, in turn, leads to the activation of phosphoinosite-3-kinase (PI3K) encoded by* age-1*, 3-phosphoinositide-dependent kinase-1 (PDK-1), AKT-1, AKT-2, and serum- and glucocorticoid-inducible kinase 1 (SGK-1). Activation of the insulin/IGF-I signalling pathway results in nuclear exclusion and thus inactivation of the forkhead transcription factor DAF-16. The heat-shock transcription factor HSF-1 and the Nrf-like xenobiotic-response factor SKN-1 are also affected by DAF-2 (reviewed in [77, 78]). Vice versa, the inhibition of the DAF-2 pathway leads to nuclear localisation and activation of the transcription factors, which changes the expression of various genes responsible for endogenous stress-response, development, metabolism, and longevity [77]. Mutations affecting components of the insulin/IGF-1 signalling pathway were found to expand the life span of nematodes by more than two times compared to wild-type worms [79, 80]. In the last years it has been found that various classes of flavonoids seem to influence this pathway [32, 36, 37, 41, 43, 45, 46, 60, 62, 69–71]. A schematic overview is shown in Figure 3.

#### 4.1.2. Influence of Flavonoids on daf-2 Expression in* C. elegans*

Xue et al. [33] studied the influence of the newly isolated quercetin derivative from onions, quercetin 3-O-*β*-D-glucopyranoside (4→1)-*β*-D-glucopyranoside (Q3M), on the gene expression of the insulin receptor* daf-2* in* C. elegans*. In this context it was shown that Q3M increased (1.38-fold)* daf-2* gene expression in the nematode compared to the control. In contrast, apigenin was found to inhibit* daf-2* expression [81].

#### 4.1.3. Influence of Flavonoids on daf-16 Gene Expression in* C. elegans*

Aspalathin (dihydrochalcone glycoside) from rooibos tea increased the* daf-16*-mRNA expression (1.2-fold) of* C. elegans* compared to untreated animals [62].

#### 4.1.4. Flavonoids Increase DAF-16 Translocation from the Cytosol into the Nucleus in* C. elegans*

The intracellular localisation of the transcription factor DAF-16 was analysed by means of transgenic* C. elegans* expressing a DAF-16::GFP fusion protein under the control of the DAF-16 promoter. Treatment with 220 *μ*M EGCG (for 1 h) resulted in in a rapid nuclear translocation of DAF-16 [69]. Analogous results were obtained by Bartholome et al. [43], where a treatment of 100 *μ*M EGCG for 48 h increased (2.2-fold) the amount of worms showing predominantly nuclear localisation of DAF-16 in contrast to untreated* C. elegans*. Similar effects were also found for quercetin [30, 36, 37], myricetin [36, 38], kaempferol, naringenin [36], fisetin [70], and apigenin [81], but not for rutin [37]. Remarkably, a 48 h pretreatment with 100 *μ*M quercetin enhanced the mean percentage of worms carrying predominantly nuclear DAF-16 by more than three times in contrast to untreated nematodes [30, 36, 37, 70].

#### 4.1.5. Flavonoid Treatment Increases Expression of DAF-16 Targets in* C. elegans*

A modulation of the DAF-16 signalling pathway can be investigated by analysing the downstream targets of DAF-16: sod-3 [82], metallothionein [83], catalase genes ctl-1 and ctl-2, gst-4 [84], and small heat shock protein (hsp) genes.

In this context it has been demonstrated that EGCG and aspalathin significantly upregulate* sod-3* mRNA expression by 70% and 320% in* C. elegans* compared to the control [62, 71]. EGCG, myricetin, kaempferol, and fisetin did also enhance the expression of SOD-3 in transgenic SOD-3::GFP* C. elegans* [36, 71]. An overview of flavonoids on DAF-16-translocation, DAF-16-mediated gene expression, and DAF-16 target protein levels is summarized in Table 5.

#### 4.1.6. Influence of Flavonoids on the Insulin/IGF-1 Signalling Investigations with Mutant* C. elegans* Strains

Using mutant nematode strains lacking the function of key genes involved in the insulin/IGF-1 signalling pathway has been an elegant way to study the potential of flavonoids to mediate stress tolerance and extend life span.

Brown et al. [60] showed that 25 *μ*M EGCG does not provoke a significant change in the intracellular ROS level of daf-16 (loss of function) mutant* C. elegans*, while in the wild-type strain ROS levels are significantly reduced by the flavonoid. This indicates that EGCG decreases ROS levels in the nematode in a DAF-16-dependent manner. Similar results were obtained with procyanidins from cocoa powder whereby DAF-16 was shown to be involved in the resistance of* C. elegans* against H_2_O_2_-induced stress [46]. Büchter et al. [38] showed that myricetin significantly decreases ROS production in wild-type nematodes for up to 6 h while in* daf-16 (mu86*) mutants a decrease was only detectable in early time points (2-3 h). Grünz et al. analysed the same compound under basal conditions and showed comparable protective effects in regard to oxidative stress levels in wild-type and* daf-16* mutant worms. This indicates that low levels of ROS as observed under short thermal stress and physiological conditions may be inactivated by direct antioxidant effects (radical scavenging) of myricetin while high concentrations of ROS can only be reduced via an indirect antioxidant mechanism. In addition, Grünz et al. [36] did also show that the quercetin and kaempferol-mediated decrease of mitochondrial ROS level did not depend on* daf-16* activity under basal conditions.

There have been conflicting results in regard to the involvement of the DAF-2 pathway in flavonoid-mediated longevity of* C. elegans*. On the one side, Pietsch et al. [32] showed that 200 *μ*M of quercetin did not extend the life span of the mutant strains* daf-2(e1368)* and* age-1(hx546) *compared to untreated controls indicating that these key genes of the insulin/IGF-1 pathway are involved in longevity regulation of* C. elegans*. A supplementary meta-analysis of global transcriptomics showed that quercetin-treated worms share transcriptional patterns with* daf-2* and* daf-12 *mutants [85] whereby DAF-12 is a nuclear hormone receptor in* C. elegans* affecting reproduction and arrest at the dauer diapause through insulin/IGF signalling [77]. Moreover, the flavonol diglycosides icariin and its conjugated form icariside II were found to mediate life span extension in a* daf-2-* and* daf-16*-dependent manner [40]. In addition catechin-induced expansion of life span seems to be in parts regulated by AKT-2 [41].

On the other hand, the life span of quercetin treated* daf-16 *mutants* (mgDf50)* was significantly increased in comparison to untreated mutant worms [32]. These results were confirmed by two other research groups, suggesting that life span extension induced by quercetin does not depend on DAF-16 activity and that the nuclear localisation of DAF-16 may not be a guaranty for longevity [36, 41]. Comparable results were obtained for myricetin by Grünz et al. [36] while we showed that the flavonoid prolonged the life span in* C. elegans* via DAF-16 [38]. These different findings may be again the result of a different experimental handling and myricetin application: agar plates versus liquid culture and different temperatures.

Catechin- and proanthocyanidins- (from blueberries) mediated longevity* in C. elegans *were also found to be independent of* daf-16 *gene expression when studying mutant strains [41, 45]. In addition catechin induced longevity seems to be independent of the* age-1* gene [41].

In conclusion it has been shown that structurally diverse flavonoids influence different regulatory components of the Insulin/IGF-1 like signalling pathway. Findings in regard to DAF-16 are inconsistent: DAF-16 translocation to the nucleus was shown for several compounds while gene expression seems to be just in parts affected. Therefore it is possible that the posttranscriptional regulation of DAF-16 is an important factor for the effects of flavonoids in* C. elegans*. Asides from that cofactors might be needed for gene expression of antioxidant enzymes and proteins. Therefore the interplay of different stress signalling pathways, with DAF-16 signalling being one of them, may result in increased stress resistance and longevity in* C. elegans*. Due to the important role of this pathway for pharmacology and toxicology, modulating effects of dietary flavonoids are getting more and more in the focus of scientific research.

### 4.2. Heat Shock Protein Response

Heat shock proteins are polypeptides protecting the cells from stress by mediating protein assembling, refolding, and translocation [86]. In* C. elegans* members of the HSP-16 protein family have been shown to be involved in thermotolerance and thus are suggested as predictors for longevity [87–89]. Further on, the expression of* hsp *genes is mainly regulated by the heat shock transcription factor-1 (HSF-1) which is also influenced by the Insulin/IGF-1 signalling pathway in* C. elegans* [88, 90]. Because of the pivotal role of HSP-16 proteins in the cellular stress response of* C. elegans* several researchers have focused on the influence of flavonoids on the expression of HSPs in this model organism. In this context HSP expression was measured by means of gene expression analysis or transgenic* C. elegans* expressing GFP under the control of various* hsp *promoters [44, 45, 62, 69, 73, 91].

#### 4.2.1. Influence of Flavonoids on hsp Gene Expression in* C. elegans*

*(1) Influence of Flavonoids on hsp Gene Expression in C. elegans: Oxidative Stress.* Oxidative stress generated by juglone has also been shown to increase HSP-16.2::GFP expression in transgenic nematodes compared to nonstressed animals. The flavonoid EGCG (220 *μ*M EGCG for 48 h) suppressed juglone-induced HSP-16.2 expression by about 73% in transgenic worms [44]. A prominent flavonoid from rooibos tea, aspalathin, showed comparable effects on the HSP level under oxidative stress [62]. In this case 20 *μ*M aspalathin (for 48 h) decreased HSP-16.2 expression by 27% after acute oxidative stress in* C. elegans* [62].

The* Gingko biloba* extract EGb 761 showed a strong capacity in decreasing HSP-16.2 expression. A posttreatment with 100 *μ*g/mL of EGb 761 significantly reduced oxidative stress-induced HSP-16.2 expression [91]. Nonetheless EGCG, aspalathin, and EGb 761 may differ in the way they interfere with HSP-16 since EGb 761 was only efficient in reducing HSP levels in transgenic* C. elegans* when posttreated to oxidative stress while EGCG and asphalatin showed significant effects when pretreated to oxidative stress [44, 91].

EGCG pretreatment reduced juglone-induced* hsp-16.1* and* hsp-16.2 *mRNA expression by about 90% in the nematodes indicating that the flavonoid is a sufficient suppressor of* hsp-16.1* and* hsp-16.2* expression on the mRNA level [69].

*(2) Influence of Flavonoids on hsp Gene Expression in C. elegans: Ageing.* The* hsp *gene expression is not only induced by cellular stress but also rises with age [92]. Therefore Wilson et al. [45] studied the effect of blueberry polyphenols on* hsp* mRNA expression in* C. elegans* between day 0 and day 4 of adulthood. The blueberry polyphenols treatment diminished the age-related increase of* hsp-12.6, -16.1, -16.49*, and -*70* mRNA expression [45].

*(3) Influence of Flavonoids on HSP::GFP Expression in Transgenic C. elegans: Thermal Stress*. Strayer et al. [91] investigated the influence of the flavonoid-rich* Ginkgo biloba* EGb 761 extract on HSP-16.2 expression in heat stressed* C. elegans*. Thermal stress caused by a temperature rise to 35°C for 2 h was shown to increase HSP-16.2 levels in transgenic nematodes compared to nonstressed animals. Subsequently, a posttreatment with 100 *μ*g/mL EGb 761 significantly suppressed stress-induced HSP-16.2 expression [91]. In contrast, Zhang et al. [71] studied the effect of EGCG on HSP-16.2 levels after transgenic* C. elegans* experienced a heat shock at 35°C. Pretreatment with 0.1 *μ*g/mL EGCG for 48 h resulted in a significant increase of HSP-16.2 expression by 11.9% in the transgenic nematodes after a 24 h recovering period of time.

### 4.3. Nrf2/SKN-1 Signalling Pathway

The nuclear factor-erythroid-2-related factor 2 (Nrf2, SKN-1* C. elegans* homologue) belongs to the basic leucine zipper (bZip) family and regulates the gene expression of phase II detoxifying enzymes and antioxidant proteins such as SOD, GST, GPO, or NAD(P)H:quinone oxidoreductase (NQO-1). Under physiological conditions in mammals, Nrf2 is bound to Keap1 which retains Nrf2 in the cytoplasm. Oxidative stress induces dissociation of the Nrf2-Keap1 complex by modification of cysteine residues of Keap1. Nrf2 translocates into the nucleus where it dimerises with other bZip transcription factors like small-Maf proteins. The heterodimer binds to antioxidant response elements (ARE) in the promoter region of genes encoding phase II enzymes and antioxidant proteins leading to a changed expression profile [93, 94].

#### 4.3.1. Flavonoids Increase SKN-1 Activation in* C. elegans*

Havermann et al. [39] demonstrated that flavonoids possess the ability to induce SKN-1 translocation into the nucleus and thus activate this transcription factor. Baicalein (100 *μ*M) increased the activation of the SKN-1 signalling pathway 4-fold [39]. In contrast the flavonoid myricetin (100 *μ*M) did not induce SKN-1 activation [38]. These two examples show that structural differences like the positions of hydroxyl groups may be critical for modulation of SKN-1 activation. Ishikado et al. [95] studied the effect of willow bark extract (WBE) containing flavonoids on the expression of the SKN-1-regulated antioxidant enzyme gamma-glutamylcysteine synthetase (gcs-1; glutamate cysteine ligase catalytic subunit (GCLC) homologue), the key enzyme for glutathione synthesis. For this purpose the researchers utilised transgenic* C. elegans* expressing GFP under the control of the* gcs-1* promoter in the pharynx and ASI chemosensory neurons under basal conditions and in the intestine under oxidative stress. Treatment with 10 mg/mL of the Willow bark extract (WBE) for 90 min increased GCS-1::GFP expression by about 19% in comparison to the control. Moreover WBE treated GSC-1::Δ2::GFP* C. elegans* lacking SKN-1-independent pharyngeal GCS-1 expression showed similar expression patterns as the GCS-1::GFP nematodes [95]. In addition transgene GCS-1::Δ2mut3::GFP animals lacking critical SKN-1 binding sites were also treated with WBE which did not result in an increased GFP expression. These experiments indicate that WBE increases gene expression of antioxidant enzymes in a SKN-1 dependent manner. The gene expression of* skn-1* seems not to be involved in catechin—and PAC (from blueberries)—mediated longevity in* C. elegans* [41, 45]. Quantitative real-time PCR analysis was conducted to measure the effect of EGCG on* skn-1*-mRNA expression in* C. elegans* and s*kn1* mRNA expression was shown to be increased in the nematode by 50% [71].

## 5. Conclusion

In the last years the model organism* C. elegans* has gained increasing importance in pharmacological and toxicological sciences due to the availability of various genetically modified nematode strains, the simplicity of modulating genes by RNAi, and the short life span. Several studies were performed demonstrating that secondary plant compounds influence ageing, stress resistance, and distinct signalling pathways in the nematode.

## Figures and Tables

**Table 1:** Modulation of the mean, median, and maximum life span in *C. elegans* by flavonoids in different studies.

Flavonoid	Concentration	Temperature	Mean	Median	Maximum	References
			life span (%)	life span (%)	life span (%)	
3′-O-Methylepicatechin	200 *μ*M	20°C	7	11	4	Surco-Laos et al., 2012 [42]
4′-O-Methylepicatechin	200 *μ*M	20°C	13	11	10	Surco-Laos et al., 2012 [42]

3-O-Methyl quercetin (isorhamnetin)	200 *μ*M	20°C	16	/	16	Surco-Laos et al., 2011 [34]
4-O-Methyl quercetin (tamarixetin)	200 *μ*M	20°C	11	/	/	Surco-Laos et al., 2011 [34]

Baicalein	100 *μ*M	25°C	45	57	14	Havermann et al., 2013 [39]

Catechin	100 *μ*M	20°C	8	11	/	Saul et al., 2009 [41]
	200 *μ*M	20°C	9	13	/	Saul et al., 2009 [41]
	200 *μ*M	20°C	No difference	No difference	No difference	Surco-Laos et al., 2012 [42]
	200 *μ*M	15°C	15	14	/	Saul et al., 2009 [41]
	200 *μ*M	23°C	11	9	/	Saul et al., 2009 [41]
	300 *μ*M	20°C	6	10	/	Saul et al., 2009 [41]
	400 *μ*M	20°C	8	11	/	Saul et al., 2011 [7]
	800 *μ*M	20°C	6	10	/	Saul et al., 2011 [7]

Epicatechin	200 *μ*M	20°C	No difference	No difference	No difference	Surco-Laos et al., 2012 [42]
	100 *μ*M	20°C	15	/	11	Bartholome et al., 2010 [43]

Epigallocatechin gallate	100 *μ*M	20°C	20	/	13	Bartholome et al., 2010 [43]
	220 *μ*M	20°C	10	/	/	Abbas and Wink, 2009 [44]
	25 *μ*M	22°C	No difference	/	No difference	Brown et al., 2006 [60]
	0,1 *μ*g/mL (=0,2 *μ*M)	20°C, 25°C	No difference	/	/	Zhang et al., 2009 [71]
	1 *μ*g/mL (=2,2 *μ*M)	20°C, 25°C	No difference	/	/	
	10 *μ*g/mL (=22 *μ*M)	20°C, 25°C	No difference	/	/	

Icariin	45 *μ*M	25°C	21	/	/	Cai et al., 2011 [40]
Icariside II	20 *μ*M	25°C	22	/	/	Cai et al., 2011 [40]

Isorhamnetin (3-O-methyl quercetin)	200 *μ*M	20°C	16	/	16	Surco-Laos et al., 2011 [34]

Kaempferol	100 *μ*M	20°C	6	/	7	Grünz et al., 2012 [36]

Myricetin	100 *μ*M	20°C	18	/	22	Grünz et al., 2012 [36]
	100 *μ*M	25°C	33			Büchter et al., 2013 [38]

Quercetin	50 *μ*M	20°C	No difference	/	/	Pietsch et al., 2011 [35]
	20 *μ*g/mL (=66,2 *μ*M)	20°C	14	/	/	Xue et al., 2011 [33]
	100 *μ*M	20°C	6	/	18	Grünz et al., 2012 [36]
	100 *μ*M	20°C	15	19	15	Kampkötter et al., 2007 [37]
	100 *μ*M	20°C	10	/	/	Saul et al., 2008 [31]
	100 *μ*M	20°C	11	/	/	Pietsch et al., 2009 [32]
	200 *μ*M	20°C	18	21	/	Pietsch et al., 2009 [32]
	200 *μ*M	20°C	10	/	/	Saul et al., 2008 [31]
	200 *μ*M	20°C	11	/	/	Surco-Laos et al., 2011 [34]
	250 *μ*M	20°C	About −7,00	/	/	Pietsch et al., 2011 [35]
Quercetin 3′-O-*β*-D-glucopyranoside	20 *μ*g/mL (=43,1 *μ*M)	20°C	12	/	/	Xue et al., 2011 [33]
Quercetin 3-O-*β*-D-glucopyranoside-(4→1)-*β*-D-glucopyranoside	20 *μ*g/mL	20°C	20	/	/	Xue et al., 2011 [33]

**Table 2:** Modulation of the mean, median, and maximum life span of *C. elegans* by flavonoid rich plant extracts in different studies.

Flavonoid rich extract	Concentration	Temperature	Mean life span (%)	Median life span (%)	Maximum life span (%)	References
Apple procyanidins	65 *μ*g/mL		12	/	/	Sunagawa et al., 2011 [47]

*Ginkgo biloba *extract EGb 761	100 *μ*g/mL	20°C	10	16	17	Kampkötter et al., 2007 [48]

Polyphenol-enriched cocoa powder	4 mg/mL	20°C	17	/	/	Martorell et al., 2011 [46]

Proanthocyanidins-enriched extract from blueberries	200 *μ*g/mL	25°C	14,4 *fem-1(hc17) *	/	/	Wilson et al., 2006 [45]

**Table 3:** Influence of flavonoids and flavonoid-rich extracts on lipofuscin levels in *C. elegans*.

Compound	Treatment/incubation time		Lipofuscin reduction (%)	References
Flavonoid: EGCG	220 *μ*M	16 days (starting one day after hatching)	39	Abbas and Wink, 2010 [69]

Fisetin	100 *μ*M	4th day of adulthood (starting from L4) (7 days old)	No difference	Kampkötter et al., 2007 [70]

Kaempferol	100 *μ*M	4th day of adulthood (starting from L4) (7 days old)	25	Kampkötter et al., 2007 [70]

Myricetin	100 *μ*M	4th day of adulthood (starting from L4) (7 days old)	33	Büchter et al., 2013 [38]

Quercetin	100 *μ*M	7th day of adulthood (3 days)	40	Surco-Laos et al., 2012 [42]
	200 *μ*M	4th day of adulthood (starting from L1) (7 days old)	16	Pietsch et al., 2011 [35]

Flavonoid rich extract: blueberry polyphenols	200 *μ*g/mL	Day 16 (starting from the first day of adulthood)	20	Wilson et al., 2006 [45]

**Table 4:** Regulatory ageing-associated genes in *C. elegans* and their mammalian homologues.

*C. elegans* gene	Mammalian homologue
*age*-1	Phosphatidylinositol 3-kinase
*akt-1, akt-2 *	Serine/threonine kinase Akt/PKB
*daf*-12	Nuclear receptor FXR
*daf*-16	FOXO transcription factor
*daf*-2	Insulin receptor, IGF-IR, IRR
*hsf-1 *	Heat shock factor 1
ins-1 bis 37	Insulin like peptide, IGF-I, IGF-II
*pdk-1 *	3-Phosphoinositide-dependent kinase 1
*skn-1 *	Nrf-2 transcription factor

*Age*: AGEing alteration; akt: AKT kinase family, *daf*: abnormal DAuer formation; FXR: farnesoid X receptor; FOXO: forkhead box O; IGF: insulin-like growth factor; Nrf: nuclear factor erythroid 2-related factor 2; skn: SKiNhead; ins: INSulin related.

**Table 5:** Modulation of gene expression and protein localisation of DAF-16 and its target SOD-3.

Effect	Flavonoid	Reference
Increased *daf-16* mRNA expression	Aspalathin	Chen et al., 2013 [62]

Nuclear translocation of DAF-16	Apigenin	Kawasaki et al., 2010 [81]
	EGCG	Abbas and Wink, 2010 [69]
		Bartholome et al., 2010 [43]
	Fisetin	Kampkötter et al., 2007 [70]
	Kaempferol	Kampkötter et al., 2007 [70]
	Myricetin	Grünz et al., 2012 [36]
		Büchter et al., 2013 [38]
	Naringenin	Grünz et al., 2012 [36]
	Quercetin	Kampkötter et al., 2007 [37]
		Kampkötter et al., 2008 [30]
		Grünz et al., 2012 [36]

Increased *sod-3* mRNA	Apigenin	Kawasaki et al., 2010 [81]
	Aspalathin	Chen et al., 2013 [62]
	EGCG	Zhang et al., 2009 [71]

Increased SOD-3 protein level	EGCG	Zhang et al., 2009 [71]
	Fisetin	Grünz et al., 2012 [36]
	Kaempferol	Grünz et al., 2012 [36]
	Myricetin	Grünz et al., 2012 [36]
